# Many human accelerated regions are developmental enhancers

**DOI:** 10.1098/rstb.2013.0025

**Published:** 2013-12-19

**Authors:** John A. Capra, Genevieve D. Erwin, Gabriel McKinsey, John L. R. Rubenstein, Katherine S. Pollard

**Affiliations:** 1 Gladstone Institutes, University of California, San Francisco, CA 94158, USA; 2 Nina Ireland Laboratory of Developmental Neurobiology, Genetics and Development, University of California, San Francisco, CA 94158, USA; 3 Bioinformatics Graduate Program, University of California, San Francisco, CA 94107, USA; 4 Institute for Human Genetics and Division of Biostatistics, University of California, San Francisco, CA 94107, USA

**Keywords:** enhancers, human accelerated regions, primate evolution, gene regulation, development

## Abstract

The genetic changes underlying the dramatic differences in form and function between humans and other primates are largely unknown, although it is clear that gene regulatory changes play an important role. To identify regulatory sequences with potentially human-specific functions, we and others used comparative genomics to find non-coding regions conserved across mammals that have acquired many sequence changes in humans since divergence from chimpanzees. These regions are good candidates for performing human-specific regulatory functions. Here, we analysed the DNA sequence, evolutionary history, histone modifications, chromatin state and transcription factor (TF) binding sites of a combined set of 2649 non-coding human accelerated regions (ncHARs) and predicted that at least 30% of them function as developmental enhancers. We prioritized the predicted ncHAR enhancers using analysis of TF binding site gain and loss, along with the functional annotations and expression patterns of nearby genes. We then tested both the human and chimpanzee sequence for 29 ncHARs in transgenic mice, and found 24 novel developmental enhancers active in both species, 17 of which had very consistent patterns of activity in specific embryonic tissues. Of these ncHAR enhancers, five drove expression patterns suggestive of different activity for the human and chimpanzee sequence at embryonic day 11.5. The changes to human non-coding DNA in these ncHAR enhancers may modify the complex patterns of gene expression necessary for proper development in a human-specific manner and are thus promising candidates for understanding the genetic basis of human-specific biology.

## 1. Introduction

Although a child can tell the difference between a chimpanzee and a human, identifying the molecular basis for the characteristics that make us human is one of the great challenges of biology. Somewhere in the millions of mutations and thousands of chromosomal rearrangements that occurred during human evolution [1,2] lie genetic changes responsible for human-specific traits, including our unique morphological features, cognitive skills, spoken language and disease susceptibilities. Two complementary types of genomic data—evolutionary signatures and functional genomics profiles—are helping researchers to narrow this search and link the specific genetic changes to molecular and organismal phenotypes.

The sequencing of genomes from many mammals, including great apes—our closest living relatives on the tree of life—and archaic hominins, enables comparative genomic analyses to quantify which parts of the human genome are conserved with other mammals and which parts distinguish us as a species. A sufficient number of genomes have been sequenced, and methods are now powerful enough to detect strong evolutionary constraint at single base pair resolution and to reliably identify moderately conserved elements the length of transcription factor (TF) binding sites [3,4]. Application of these methods to whole genome multiple sequence alignments revealed that 5–10% of the human genome is conserved across mammals, most of which is not in protein-coding regions [4–7]. These discoveries indicate that the non-coding portion of the human genome harbours more functionally constrained DNA than the coding portion, making it a larger potential target for evolutionary change. Supporting this idea, the catalogue of protein-coding changes that happened during human evolution is too small to explain all of our unique traits [1,8,9]. Thus, comparative genomics shows that many human phenotypes likely result from changes to regulatory elements, as originally hypothesized by King & Wilson [10] and consistent with evidence from other animals [11] and human population genetic studies [12].

Phylogenetic analysis of mammalian genomes also provides specific evidence for the locations of human-specific regulatory elements in the vast non-coding portion of the genome. Many highly conserved sequences function as gene regulatory enhancers [13]. Based on this idea, several groups developed computational approaches to scan mammalian sequence alignments for evolutionarily conserved sequences that have changed significantly in humans since divergence from chimpanzees (or archaic hominins). For example, using phyloP, a method based on likelihood ratio tests for accelerated sequence divergence on the human lineage [3,14], we previously identified 721 human accelerated regions (HARs) [4,15]. This approach does not constrain HARs to be non-coding, yet 92% of them are, underscoring the likely importance of regulatory sequences in recent human genome evolution. Other groups focused specifically on conserved non-coding elements and developed methods to identify those with the most sequence changes in humans [16–18]. Together, these approaches have identified 2649 unique non-coding HARs, which we will collectively refer to as ncHARs (see §2).

The primary challenge in associating ncHARs with changes in human gene regulation lies in the fact that the vast majority of these genomic elements lie in uncharacterized regions of the human genome and their evolutionary conservation does not predict a specific function. Efforts to experimentally characterize the ncHARs with the most sequence changes in humans have revealed that HAR1 is a novel RNA gene expressed during development of the neocortex [19], and HAR2 (also known as HACNS1) is a human-specific developmental enhancer [20]. Several human genes are associated with clusters of multiple ncHARs. A recent study showed that 11 of 14 ncHARs located within the *NPAS3* locus are active enhancers during brain development [21], at least one of which has human-specific enhancer activity [22]. These findings demonstrate that ncHARs are a rich collection of candidates to search for the genetic basis of human-specific gene regulation. But the low-throughput and high cost of functional assays, coupled with the large number of ncHARs, requires that we develop automated procedures to prioritize candidates for experimental characterization.

Functional genomics provides valuable data for predicting which ncHARs function as regulatory elements. As exemplified by the ENCODE Project [23], the functional genomics approach uses a collection of high-throughput experimental procedures that leverage next-generation sequencing to functionally annotate different cell types from an organism. For example, RNA-sequencing (RNA-seq), measurements of open chromatin, and chromatin immunoprecipitation followed by sequencing (ChIP-seq) with antibodies to RNA polymerase, TFs and specific histone modifications, can identify actively transcribed genes and their promoters [23–25], as well as distal enhancers [26,27]. Additionally, computational approaches that integrate many functional genomics datasets can be applied to segment the human genome into functional classes, including several types of regulatory regions [28–30]. These results suggest that it may be possible to identify ncHARs with a particular function by integrating sequence data and functional genomics data.

In this paper, we focus on predicting and validating ncHARs that function as developmental enhancers. We chose this problem for several theoretical and practical reasons. First, the exquisite control of gene expression in embryonic development suggests changes in spatial or temporal activity of ncHAR enhancers could lead to major phenotypic effects. Second, many ncHARs are associated with developmentally expressed genes and enhancer-associated epigenetic marks. Third, it is possible to assay enhancer activity of human sequences in whole mouse embryos, as was done to characterize HAR2 and the *NPAS3* locus. Finally, many well-defined developmental cell types can be sorted by molecular markers, facilitating downstream functional studies to investigate consequences of enhancer variants. Our approach combines machine-learning techniques for predicting developmental enhancers, analyses of ncHAR sequences to identify human mutations that are most likely to have altered enhancer activity, and enhancer assays to test our predictions. We show that many ncHARs function as developmental enhancers, and suggest that the human and chimpanzee versions of some of these enhancers drive different expression patterns in embryonic development.

## 2. Results

### (a) Thousands of non-coding loci exhibit accelerated substitution rates in the human lineage

**Figure 1.**
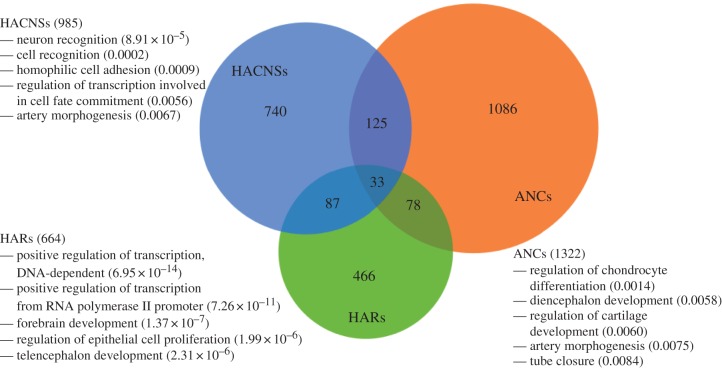
Overlap of different sets of non-coding human accelerated regions and their top enriched gene ontology (GO) biological process annotations. The independently defined sets of ncHARs considered in this study display only modest overlap. However, the functional annotations enriched in nearby genes compared with the genomic background share common themes of development (e.g. differentiation, proliferation and morphogenesis) and regulation. The GO biological process annotations enriched when all ncHARs are considered together show similar general patterns (table 1). The full sets of enriched GO biological process annotations for each set are given in electronic supplementary material, table S1. ‘HACNSs’ are human accelerated conserved non-coding sequences [17]; ‘ANCs’ are accelerated conserved non-coding sequences [16]; and ‘HARs’ are the non-coding subset of the HARs [4,15]. The 63 accelerated regions from Bush & Lahn [18] are not pictured here to aid clarity, and because they lacked significant GO biological process enrichment. (Online version in colour.)

In the following analyses, we consider four sets of HARs: 721 HARs obtained by merging those defined by similar methods in Pollard *et al.* [15] and Lindblad-Toh *et al.* [4]; 992 human accelerated conserved non-coding sequences (HACNSs) defined by Prabhakar *et al.* [17]; 1356 accelerated non-coding conserved sequences (ANCs) from Bird *et al.* [16]; and 63 accelerated elements from Bush *et al.* [18]. These sets have some regions in common, but the majority of accelerated regions are identified by only one study (figure 1). This is not entirely surprising given that, despite having similar goals, each set was defined using different statistical tests, filters and multiple species alignments (see §§3 and 4 for more details).

By definition, three of these four accelerated region sets contain only non-coding sequences; the exception is the HARs, which were defined in a scan of both coding and non-coding conserved elements. We analysed the distribution of the 721 HARs with respect to human genes from GENCODE (v. 14) and found that only 57 (8%) overlap an annotated coding region compared with 20% of the filtered mammal conserved elements from which they were identified. This significant enrichment of HARs in non-coding regions strongly supports the hypothesis that regulatory change has been important in recent human evolution (see §1). We note that seven of the 992 HACNSs and 34 of the 1356 ANCs overlap coding regions; these likely reflect changes in gene annotations over the past several years.

**Figure 2.**
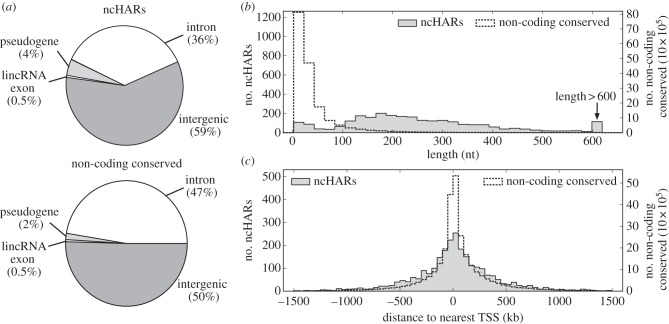
Genomic distribution of ncHARs compared with non-coding conserved elements. (*a*) The genomic distribution of ncHARs demonstrates that the vast majority are found in unannotated intronic or intergenic regions. ncHARs are more likely to be found in pseudogenes and intergenic regions than non-coding evolutionarily conserved elements. ncHARs overlapping pseudogenes may have an accelerated substitution rate on the human lineage owing to loss of negative selection. (*b*) The ncHARs are relatively short, with an average length of 257 nt and only seven regions longer than 1 kb. The non-coding conserved regions are significantly shorter than the ncHARs (*p* ≈ 0, Mann–Whitney *U*-test (MWU)), but this difference is likely driven by the greater power to detect recent acceleration in longer conserved elements. All non-coding conserved elements shorter than the shortest ncHAR (13 nt) were not considered in this plot. (*c*) ncHARs are found at a range of distances and orientations from the nearest TSS, with many more than 100 kb away and a few as distant as 2 Mb. The ncHARs are more distant from the nearest TSS than non-coding conserved regions (*p* ≈ 0, MWU test).

To produce a comprehensive list of non-coding human accelerated regions (ncHARs), we combined all accelerated regions from these five studies, merged overlapping regions, and removed any regions that overlap a protein coding sequence. This resulted in a collection of 2649 unique ncHARs. Of these ncHARs, 962 (36%) are intronic; 117 (4%) overlap a pseudogene; 15 (0.5%) overlap an exon of a lincRNA, and none overlap a known miRNA. The remaining 59% are intergenic (figure 2*a*). The ncHARs are quite short, with an average length of 257 nucleotides (nt) and a standard deviation of 171 nt (figure 2*b*). The ncHARs display a wide range of distances to the nearest transcription start site (TSS); the average distance is about 307 kilobases (kb) with a standard deviation of 342 kb (figure 2*c*). Both the length and TSS distance distributions for ncHARs are significantly different from those of mammalian conserved non-coding elements (*p* ≈ 0 for both, Mann–Whitney *U*-test; figure 2*b*,*c*).

To explore potential functions for the ncHARs, we associated each ncHAR with nearby genes and analysed the genes’ annotations [31]. We found significant enrichment for functions involved in development and regulation among the genes near the ncHARs (table 1). Different specific biological process terms were enriched for the sets of ncHARs from the different studies (see electronic supplementary material, table S1), but developmental processes were highly enriched within each set compared with the genomic background (figure 1). By contrast, no biological process terms were significantly enriched among the ncHARs when compared with a background of the conserved non-coding elements. In all, 1385 ncHARs (52%) are located within 1 megabase (Mb) of a developmental gene, which could potentially be differentially regulated in humans owing to sequence changes in the ncHAR. These findings suggest that functions in development, as demonstrated in the experimental characterizations of HAR1, HAR2 and the *NPAS3* locus, are likely common among the ncHARs.

**Table 1.** Enriched GO biological process annotations in genes near ncHARs compared with the genomic background. The terms are almost universally associated with regulation of development, particularly of the brain.

GO term	*Q*-value	no. ncHARs
regulation of epithelial cell proliferation involved in lung morphogenesis	3.2 × 10^−8^	27
neuron recognition	2.9 × 10^−6^	61
regulation of chondrocyte differentiation	5.9 × 10^−6^	51
adherens junction organization	8.2 × 10^−6^	64
regulation of transcription involved in cell fate commitment	2.9 × 10^−5^	23
artery morphogenesis	3.8 × 10^−5^	35
smooth muscle tissue development	3.8 × 10^−5^	33
positive regulation of epithelial cell proliferation involved in lung morphogenesis	4.7 × 10^−5^	19
regulation of cartilage development	5.2 × 10^−5^	57
cell proliferation in forebrain	0.00024	29
artery development	0.00035	36
positive regulation of myoblast differentiation	0.00037	25
neural precursor cell proliferation	0.0018	42
regulation of myoblast differentiation	0.0032	31
axon choice point recognition	0.0063	21
axon midline choice point recognition	0.0070	18
olfactory bulb interneuron differentiation	0.0070	25
central nervous system projection neuron axonogenesis	0.0073	24
olfactory bulb interneuron development	0.0077	22
negative chemotaxis	0.010	30
lacrimal gland development	0.011	12
ventral spinal cord development	0.011	31
keratinocyte proliferation	0.016	16
positive regulation of filopodium assembly	0.016	13
regulation of transcription from RNA polymerase II promoter involved in ventral spinal cord interneuron specification	0.022	9
regulation of transcription from RNA polymerase II promoter involved in spinal cord motor neuron fate specification	0.025	8
mammary gland specification	0.030	12
specification of organ identity	0.036	20
regulation of filopodium assembly	0.036	16
positive regulation of cartilage development	0.046	21
spinal cord motor neuron cell fate specification	0.049	12

Supporting the hypothesis that mutations in ncHARs alter the expression of nearby genes in humans, 1575 (59%) of ncHARs are within 1 Mb of a gene differentially expressed between human and chimpanzee in at least one context [32,33]. Interestingly, 1377 (52%) ncHARs are within 1 Mb of at least one TF, and 492 (19%) have a TF as their closest gene. So, ncHARs have the potential to both directly and indirectly influence the expression of many genes.

### (b) Functional genomics data suggest functions for many non-coding human accelerated regions

Recent advances in experimental and DNA sequencing technology have enabled the collection of genome-wide data that are informative about the function of non-coding regions in different cell types [23]. We analysed the ncHARs in the context of a recent segmentation of the non-coding genome into functional domains based on unsupervised clustering of data generated by ENCODE [30]. This analysis summarized data from six of the ENCODE cell lines: GM12878, H1-ESC, HUVEC, HeLa-S3, HepG2, K562. The majority of ncHARs are in predicted low activity regions in these cell types; however, many are found in states likely to have regulatory functions in some cell lines. There are 285 ncHARs in a predicted enhancer domain in at least one cell type; 82 are in promoter domains; and 79 are in regions enriched for CTCF, binding which can indicate physical looping of distal enhancers to their promoters as well as insulator functions. This suggests that the ncHARs may be involved in both promoting and repressing gene expression. However, this analysis is limited by the availability of regulatory annotations to only six cell types, none of which, with the possible exception of H1-ESC cells, are directly relevant to development.

To consider additional cellular contexts beyond the six cell lines with predefined segmentations, we analysed ChIP-seq data for enhancer-associated histone modifications (H3K4me1 and H3K27ac) and a transcriptional coactivator (P300/CBP) from several additional ENCODE cell lines (§4; see electronic supplementary material, table S2). Of the 2649 ncHARs, 426 overlap a P300 site; 1026 overlap a H3K27ac peak; and 1331 overlap a H3K4me1 peak in at least one cellular context. Altogether, nearly 60% (1571) of the ncHARs overlap at least one of these common markers of enhancer activity in at least one context.

### (c) Diverse datasets predict that several hundred non-coding human accelerated regions are developmental enhancers

To focus our analysis on embryonic development and integrate data about known biologically active enhancers, we applied EnhancerFinder, a developmental regulatory enhancer prediction method that we recently developed, to the ncHARs. EnhancerFinder is a machine-learning algorithm that integrates DNA sequence, evolutionary patterns and functional genomics data from many cellular contexts to accurately predict developmental enhancers of gene expression [34]. The algorithm is trained and evaluated on a set of nearly 1500 human sequences experimentally tested for developmental enhancer activity taken from the VISTA Enhancer Browser [35].

**Figure 3.**
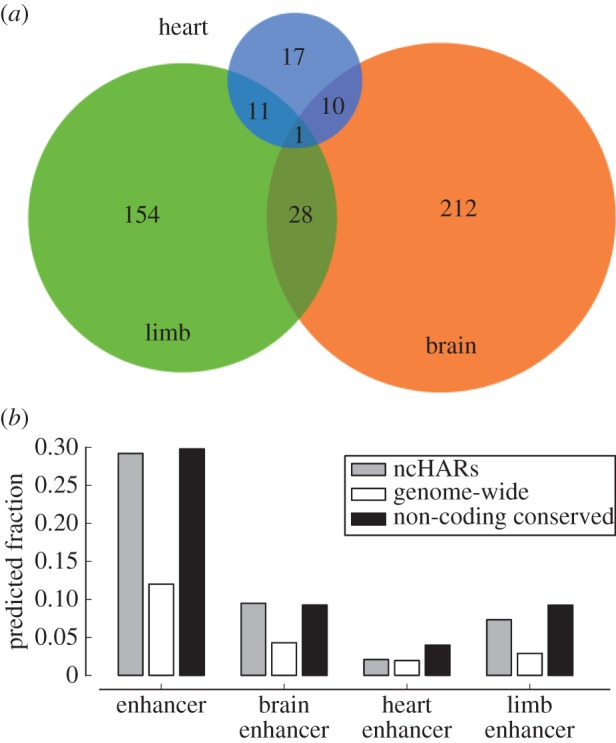
Predicted ncHAR enhancers and their tissues of activity. (*a*) We applied the EnhancerFinder enhancer prediction pipeline to the 2649 ncHARs; 773 were predicted to be developmental enhancers. Among this set, EnhancerFinder predicted 251 brain enhancers, 194 limb enhancers and 39 heart enhancers. (*b*) We compared the predicted fraction of enhancers and tissue-specific enhancers between the ncHARs, the genome-wide non-coding background, and a filtered set of non-coding conserved regions (mammalian phastCons elements). The ncHARs are dramatically enriched for predicted enhancer, brain enhancer, and limb enhancer activity compared with the genomic background; however, they are not significantly different from the non-coding conserved regions. Supporting these predictions, validated ncHAR developmental enhancers (table 2 and electronic supplementary material, table S4) show a strong enrichment for brain activity, though this may reflect biases in how they were selected for experimental analysis. The relative lack of heart enhancers may reflect the fact that this tissue follows an earlier developmental trajectory than brain and limb. (Online version in colour.)

EnhancerFinder predicts that 773 of the 2649 ncHARs (29%) are human developmental enhancers (see electronic supplementary material, table S3). This is significant enrichment for enhancer activity; EnhancerFinder predicts that approximately 12% of all non-coding regions genome-wide are likely to have developmental enhancer activity (figure 3*b*). However, the fraction of ncHARs that EnhancerFinder predicts to be human enhancers is quite similar to the fraction of conserved non-coding elements predicted to function as enhancers by EnhancerFinder (approx. 30%). These results suggest that many conserved regions, including ncHARs, function as developmental enhancers. Because some ncHARs may have lost enhancer function specifically in the human lineage [21], we estimate that at least one-third of ncHARs function as developmental enhancers in mammals. However, as suggested in §2*b*, the fraction may be even higher, and some ncHARs likely function as enhancers in adult cell types.

EnhancerFinder can also predict tissues in which enhancers are likely to be active. We have sufficient data to accurately assign predicted enhancers to brain, limb and heart activity domains [34]. We predict that 251 of the 773 ncHAR enhancer candidates are active in brain development, 194 are active in limb development and 39 are active in heart development (figure 3*a*). While the majority of ncHAR enhancers are predicted to be active in only one of these tissues, 50 are assigned to two tissues, and one to all three tissues. Interestingly, the numbers of ncHARs predicted to be brain and limb enhancers are each significantly more than expected from our genome-wide analysis (figure 3*b*). We could not make a confident tissue assignment for the remaining 333 predicted ncHAR enhancers, either because they did not meet the cut-off we used for assignment to brain, heart or limb, or because they are likely active in other embryonic tissues. All of the predicted ncHAR enhancers are promising regions for further investigation of the genetic basis for human-specific traits.

### (d) Transcription factor binding site differences between human and chimpanzee pinpoint non-coding human accelerated region enhancers that may function uniquely in humans

Regulatory sequences often tolerate significant change while maintaining their functions [36,37], yet a single enhancer mutation can yield disease and severe phenotypes [38]. The significant acceleration in substitution rate observed along the human branch in the ncHARs provides strong evidence for a human-specific change in function at these loci. However, it is not clear from evolutionary patterns alone which mutations are likely to influence regulatory function and which are likely to be silent.

To prioritize the potential ncHAR enhancers for further study, we analysed the divergence of predicted transcription factor binding sites (TFBS) between the human and chimpanzee sequences in the hope of gaining additional evidence of functional change. A recent cross-species analysis of developmental enhancers demonstrated that divergence in the number of predicted TFBS better predicts changes in enhancer activity between species than does overall sequence divergence [39]. Using motif models for 220 TFs from the vertebrate non-redundant set in TRANSFAC, we applied the binding site divergence model to the human and chimpanzee sequences of ncHARs. Each ncHAR exhibits differences in the number of predicted binding sites for many TFs between the human and chimpanzee sequences. Overall, the divergence is significant for an average of 2.17 TFs between human and chimpanzee, and 57% of the ncHARs show significant divergence for at least one TF (*p* < 0.1). To further characterize and prioritize the ncHARs with TFBS divergence between human and chimpanzee, we also manually analysed the annotations and expression patterns of nearby genes.

### (e) Many non-coding human accelerated regions function as enhancers during embryonic development

The ability of a DNA sequence to enhance the expression of a gene *in vivo* can be tested using transgenic assays in model organisms, such as mouse, fly and zebrafish. In these experiments, a construct is built with the sequence of interest placed upstream of a minimal promoter and a reporter gene, such as *LacZ*. The construct is injected into fertilized eggs, and transgenic individuals are assayed for reporter gene expression at a relevant time point. Nearly 1900 human and mouse DNA sequences have been assayed for enhancer activity in transgenic mice at embryonic day 11.5 (E11.5) and made available in the VISTA Enhancer Browser [35,40].

Twenty-three of the ncHARs overlap a validated enhancer sequence from VISTA (table 2), and an additional 24 ncHARs overlap VISTA negative regions. Brain enhancers are the most common among the validated enhancers, with 16 instances, but there are also ncHAR enhancers active in the neural tube, limb, heart, facial mesenchyme, eye and ear. These results add additional support to our prediction that many ncHARs are developmental enhancers. But because there are very few regions in VISTA for which both the human and mouse (ancestral) sequences have been tested, and no chimpanzee sequences have been tested, they are not informative about their importance in driving human-specific regulatory patterns.

**Table 2.** Many ncHARs overlap regions with enhancer activity. The VISTA Enhancer Browser has tested the ability of nearly 1900 human and mouse sequences to drive expression in E11.5 transient transgenic mice. The 23 listed ncHARs overlap a positive element from VISTA. Aliases for elements discovered in more than one study are given in footnotes.

ncHAR	VISTA ID	active tissues	genes within 1 Mb
ANC162	hs169	neural tube	ATG4C, DOCK7, FOXD3, ALG6, ANGPTL3, EFCAB7, DLEU2L, USP1, PGM1, ITGB3BP, KANK4, ROR1, L1TD1
HAR164	hs1198	other	PKN2, LMO4, GTF2B, CCBL2, RBMXL1, GBP3, GBP1
2xHAR.97	hs878	limb, tail	PKN2, GTF2B, LMO4, CCBL2, RBMXL1, GBP3, GBP1, GBP2
ANC494	hs327	hindbrain	PKN2, GTF2B, CCBL2, RBMXL1, GBP3, GBP1, GBP2, GBP7, GBP4, GBP5, GBP6
ANC547	hs1301	forebrain	C11orf58, SOX6, PLEKHA7, RPS13, PIK3C2A, NUCB2, NCR3LG1, KCNJ11
2xHAR.514^a^	hs234	midbrain, eye	RCN1, PAX6, ELP4, IMMP1L, DNAJC24, DCDC1, DCDC5, WT1, EIF3M
2xHAR.447	hs798	neural tube, forebrain	DERA, STRAP, EPS8, SLC15A5, MGST1, LMO3, RERG, PTPRO
HACNS567	hs882	neural tube, hindbrain, midbrain, dorsal root ganglion, trigeminal	KLHL1, DACH1
ANC86	hs828	hindbrain	C15orf41, MEIS2
ANC806	hs634	neural tube, hindbrain, midbrain, forebrain	SALL3, ATP9B
HAR122^b^	hs1181	heart	FANCL
2xHAR.238^c^	hs522	hindbrain, forebrain, other	TFCP2L1, GLI2, CLASP1, MKI67IP, TSN, INHBB, RALB, TMEM185B
HAR2^d^	hs521	limb, branchial arch, eye, ear	AC064874.1, AC079135.1, GBX2, AGAP1, ASB18, IQCA1, CXCR7, SH3BP4
HAR104^e^	hs526	forebrain	FAM53A, SLBP, TMEM129, TACC3, FGFR3, NKX1-1, CRIPAK, LETM1, WHSC1, UVSSA, MAEA, CTBP1, SPON2, AC092535.1, WHSC2, C4orf48, NAT8L, RNF212, FGFRL1, SLC26A1, POLN, HAUS3, IDUA, DGKQ, MXD4, TMEM175, GAK, CPLX1, ZFYVE28, RP11-503N18.3, RNF4, PCGF3, MFSD7, ATP5I, MYL5, PDE6B
ANC1061	hs847	forebrain	BEND4, SLC30A9, DCAF4L1, TMEM33, SHISA3, PHOX2B, ATP8A1, GRXCR1, LIMCH1, UCHL1, APBB2
ANC55^f^	hs1175	forebrain, nose	ID4, MBOAT1, E2F3, CDKAL1
HACNS21	hs1366	midbrain	BTBD9, GLO1, DNAH8, ZFAND3, GLP1R, MDGA1, SAYSD1, KCNK5, CCDC167, KCNK17, KCNK16, FTSJD2
HACNS584	hs1738	limb	GRIK2, ASCC3, SIM1
HAR143	hs1809	neural tube, hindbrain, midbrain, forebrain	FEZF1, AASS, RNF133, RNF148, PTPRZ1, CADPS2, TAS2R16, SLC13A1, FAM3C
HAR118^g^	hs669	midbrain	RUNX1T1, SLC26A7, LRRC69, OTUD6B, TMEM55A, RP11-122A3.2
HAR34	hs852	forebrain, facial mesenchyme	LINC00583, MPDZ, NFIB, ZDHHC21, CER1, LURAP1L
ANC1335	hs123	forebrain	ARX, POLA1, PCYT1B, MAGEB18, MAGEB6, MAGEB5, PDK3
2xHAR.393	mm170	heart	IQGAP1, ZNF774, GABARAPL3, CRTC3, NGRN, RP11-697E2.6, TTLL13, CIB1, GDPGP1, SEMA4B, IDH2, BLM, ZNF710, FURIN, FES, C15orf38, C15orf38-AP3S2, MAN2A2, AP3S2, UNC45A, HDDC3, RCCD1, ANPEP, PRC1, VPS33B, MESP2, MESP1, SV2B, WDR93, PEX11A, PLIN1, KIF7, TICRR, RHCG

^a^ANC553; ^b^HACNS851; ^c^BUSH35 HACNS4; ^d^ANC6, BUSH38, HACNS1, 2xHAR.3; ^e^HACNS33, ANC107; ^f^HACNS549; ^g^HACNS126.

To identify additional ncHAR enhancers, evaluate our EnhancerFinder algorithm, and enable comparisons of cross-species activity, we performed enhancer assays in transgenic mice for 30 uncharacterized ncHARs. These include one negative control (HAR69), which showed no evidence of being a developmental enhancer, plus 29 ncHARs with EnhancerFinder scores suggestive of developmental enhancer activity (13, brain; 8, limb; 7, neural tube; 4, heart and 8 other tissues). The 29 ncHAR enhancer candidates are also associated with developmental genes, and the human substitutions in their sequences create and destroy predicted TFBS.

Our experiments found suggestive developmental enhancer activity at E11.5 for 24 of 29 (83%) predicted ncHAR enhancers (see electronic supplementary material, table S4 and figure S1). For 17 of these 24, the expression patterns within the major domains of activity (e.g. forebrain, limb), were highly consistent and reproducible. Additionally, 16 of the 24 tested ncHARs with EnhancerFinder predictions of tissue specificity showed enhancer activity in at least one of the predicted tissues. The negative control showed no consistent activity in any tissue. We also note that some enhancer activity was observed for several of the negatives (see electronic supplementary material, figure S1), so it is possible that consistent patterns would emerge for these sequences if more transgenic embryos were generated at E11.5 or other developmental stages. In addition to the electronic supplementary material, a searchable database of images of all transgenic mouse embryos generated in this study is freely available online at http://lighthouse.ucsf.edu/enhancerbrowser/.

### (f) Several non-coding human accelerated regions exhibit enhancer activity suggestive of human–chimpanzee differences

**Figure 4.**
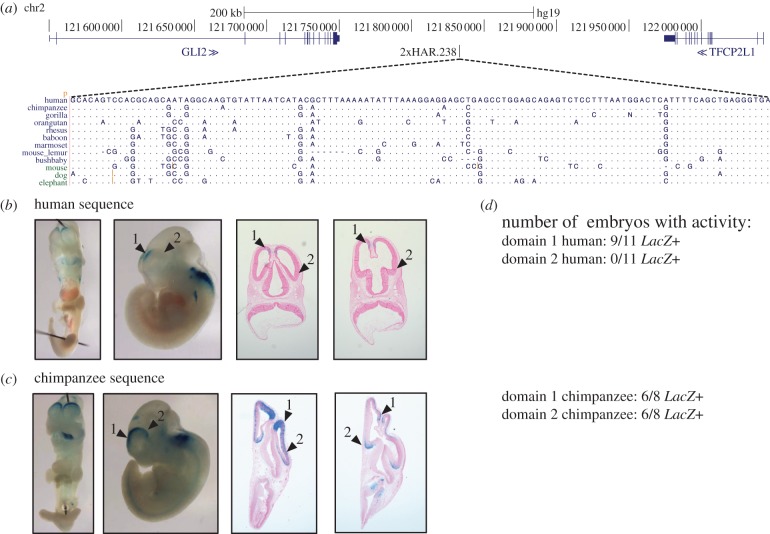
The 2xHAR.238 enhancer drives activity patterns in transgenic mice suggestive of brain expression differences between human and chimpanzee. (*a*) 2xHAR.238 is located on chromosome 2 and flanked by GLI2 and TFCP2L1. The alignment illustrates the human-specific substitutions in the ncHAR; bases matching the human sequence are shown as dots. A kilobase of sequence surrounding the ncHAR was cloned into a *LacZ* reporter construct. (*b*,*c*) One representative E11.5 transgenic mouse embryo is shown in two whole mount views plus two cross sections for the human sequence (*b*) and chimpanzee sequence (*c*). Both constructs produce consistent *LacZ* staining (blue) in the rostral dorsal pallium (arrowhead 1), the dorsal part of caudal hindbrain, and the rostral spinal cord. A second, more caudal, part of the dorsal pallium (arrowhead 2) shows activity unique to the chimpanzee sequence. The staining in the sectioned embryos shows human and chimpanzee enhancer activity in progenitor cells of the rostral dorsal pallium (domain 1), whereas only the chimpanzee enhancer has activity in the caudal dorsal pallium (progenitor cells and neurons; domain 2). The flanking gene, GLI2, is expressed in the cortex at E11.5 in mouse, and is thus a promising candidate target. (*d*) The activity patterns illustrated by the example images are consistent across embryos. All embryo images are given in electronic supplementary material, figure S1.

**Figure 5.**
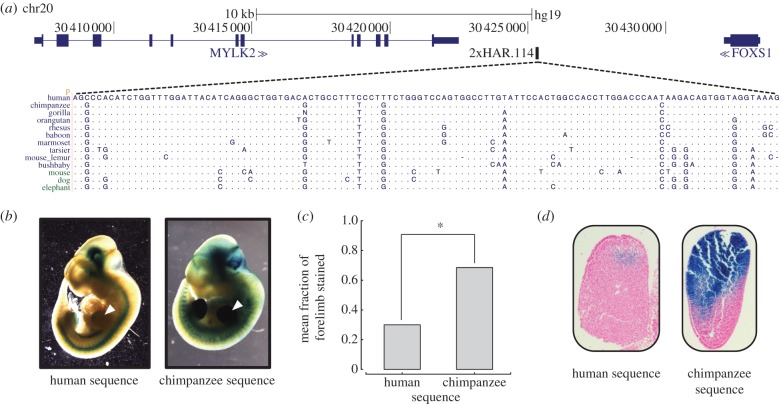
The human and chimpanzee sequences for 2xHAR.114 drive different activity patterns in the developing limbs of transgenic mice. (*a*) 2xHAR.114 is located on chromosome 20 and flanked by MYLK2 and FOXS1. The organization and details of this figure are the same as in figure 4. (*b*) Both the human and chimpanzee sequence produce consistent staining in the limb (white triangles) and neural tube, as well as suggestive staining in the brain. The flanking genes are known to be involved in heart development. Additional embryo images are given in electronic supplementary material, figure S1. (*c*) The chimpanzee sequence consistently drives more extensive activity in the limb at E11.5. The mean fraction of the forelimb stained across all *LacZ* positive mouse embryos with the human construct was significantly lower than with the chimpanzee construct (*p* = 0.004; *t*-test). (*d*) Cross sections of mouse embryonic forelimbs showing the patterns of *LacZ* expression (blue) driven by the human and chimpanzee 2xHAR.114 enhancers. Both enhancers have limb mesenchyme activity, but the chimpanzee enhancer has a much larger domain of activity.

**Figure 6.**
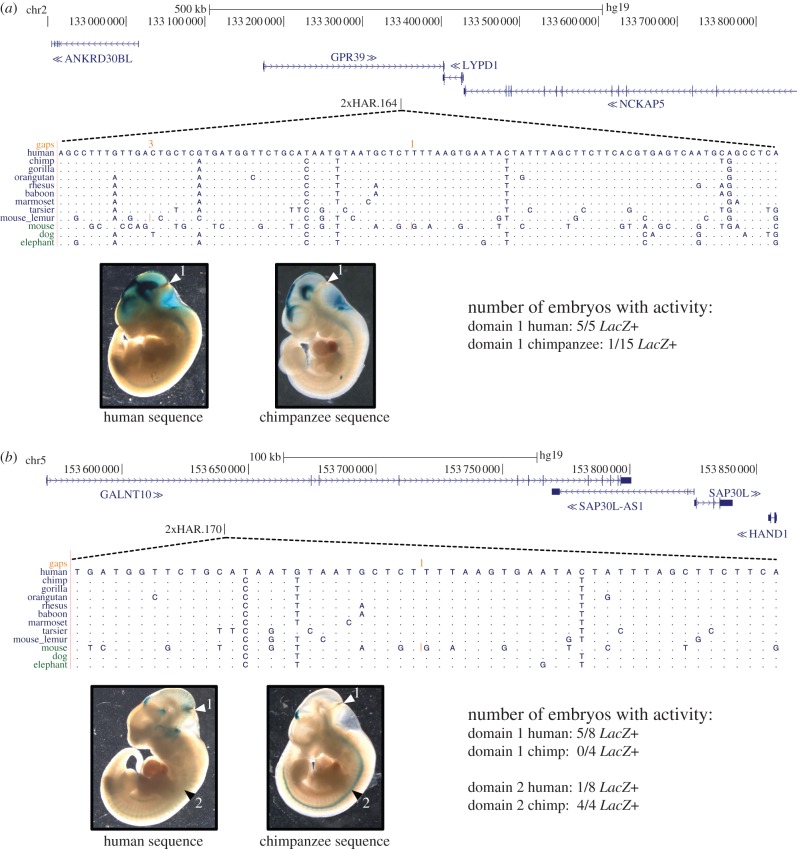
Two ncHARs drive patterns suggestive of unique brain expression in human at the midbrain–hindbrain boundary in transgenic mice. The components of this figure are the same as in figure 4. (*a*) The genomic context, sequence alignment, and activity domains driven by human and chimpanzee 2xHAR.164 in E11.5 transgenic mice. Nearby developmental genes include LYPD1 and NCKAP5. Both human and chimpanzee sequences drive consistent activity in several brain structures, including the dorsal telencephalon, dorsal pretectum, roof plate of the diencephalon and midbrain, ventral diencephalon, midbrain and hindbrain. However, expression in the boundary between the midbrain and hindbrain (isthmus) is human-specific (arrowhead 1). The mouse orthologue of the nearby LYPD1 gene is expressed in the midbrain at E11.5. (*b*) 2xHAR.170 also produces human-specific activity in the isthmus (arrowhead 1). In addition, the chimpanzee construct drives strong spinal cord expression, whereas the human construct does not (arrowhead 2). The developmental gene HAND1 is a potential target gene of the 2xHAR.170 enhancer. All embryo images are given in the electronic supplementary material, figure S1.

We tested each candidate ncHAR enhancer using both the human and chimpanzee reference genome sequence. The chimpanzee sequence is likely representative of the ancestral state, because all HARs are highly conserved across non-human mammals (see §3). Comparing the *LacZ* staining patterns associated with the human versus chimpanzee sequences revealed suggestive inter-species differences in enhancer activity for five of the 17 ncHARs with consistent developmental enhancer activity in E11.5 embryos. For example, 2xHAR.238 drives consistent staining in the forebrain (rostral dorsal pallium), the dorsal part of the caudal hindbrain, and the rostral spinal cord. A second, more caudal, part of the dorsal pallium shows activity unique to the chimpanzee sequence (figure 4). Similarly, for 2xHAR.114, both the human and chimpanzee sequence drive consistent expression patterns in the developing limb, but the activity domain of the chimpanzee sequence is significantly broader (*p* = 0.004; *t*-test) than that of the human sequence (figure 5). In contrast to the reduction of human activity in these two examples, the human sequences for two other ncHARs drive more extensive expression patterns in the developing brain than their chimpanzee counterparts. 2xHAR.164 and 2xHAR.170 drive activity in many regions of the brain, and both exhibit activity at the midbrain–hindbrain boundary (isthmus) that is unique to the human sequence at E11.5 (figure 6). For 2xHAR.170, the chimpanzee sequence also consistently produces strong neural tube expression, whereas the human sequence does not. Finally, the human sequence of HAR25 yields weak, but consistent, expression in the developing eye that is not observed with the chimpanzee sequence (see electronic supplementary material, figure S2).

## 3. Discussion

In this paper, we used bioinformatics and *in vivo* assays to explore the hypothesis that many ncHARs function as developmental enhancers. We found strong support for this hypothesis from a number of complementary lines of evidence. First, we examined the genomic distribution of HARs (a mix of coding and non-coding regions [4,15]) and showed that they are enriched in intronic and intergenic regions of the human genome compared with conserved elements. Second, we compiled a collection of 2649 ncHARs (specifically non-coding) from a variety of comparative genomics studies. Despite the fact that each of these studies used different methods and sequence alignments and therefore identified many ncHARs that were not found in the other studies, the ncHARs from all four studies are consistently enriched nearby genes with annotated functions in embryonic development (figure 1 and table 1). However, we note that while the distribution of ncHARs across the genome is somewhat different from all conserved non-coding elements (figure 2), we found no significant differences in the biological process annotations of nearby genes. Thus, many ncHARs are likely to function as developmental enhancers, but so do a similar fraction of all conserved non-coding elements.

Next, we applied genome segmentation and enhancer prediction methods, both based on functional genomics data, to explore indirect experimental evidence that ncHARs are located in regulatory regions of the human genome. More than half of the ncHARs showed evidence of enhancer activity in at least one cellular context. We observed a significant enrichment of ncHARs among predicted developmental enhancers, particularly those predicted to be active in the embryonic brain and limb. Additionally, more than 100 ncHARs are predicted to be in promoters and other types of regulatory elements. Finally, we used associations with developmental genes and predicted losses and gains of TFBSs to select 29 ncHAR enhancer candidates to validate using reporter gene based enhancer assays in transgenic mice. We discovered 24 enhancers that are active at E11.5 in various embryonic tissues, five of which suggest different expression patterns between human and chimpanzee.

Combining our results with those from two previous studies of ncHARs [20,21] and the VISTA Enhancer Browser [35], we conclude that 56 of 88 ncHARs tested to date (64%) are developmental enhancers. Extrapolating our findings to the full set of 2649 ncHARs, we estimate that more than 1600 are developmental enhancers. The true number may, however, be lower because the ncHARs that have been validated were not selected randomly. On the other hand, transient transgenic enhancer assays sometimes fail to identify true enhancers and generally examine only a single developmental stage (E11.5 in most of these assays). The similarity of this estimate to the fraction of ncHARs with evidence of enhancer activity in functional genomics data from ENCODE suggests that the true number could be this high.

Documenting divergent enhancer activity and linking this to gene expression changes is challenging for a number of reasons, including the inherent noisiness of enhancer assays and the possibility that human-specific enhancer activities may be subtle, quantitative and restricted to specific cells and/or developmental time points. Previous work identified tissue-specific enhancer activity unique to the human ncHAR sequences of HAR2 [20] (see also [41–43]) and 2xHAR.142 [22].

Many of the cellular, histological and morphological differences between humans and chimpanzees are likely to be established later in development than the relatively evolutionarily conserved stage we tested, E11.5. Nonetheless, several ncHAR enhancers drive expression patterns suggestive of differences in humans compared with chimpanzees. In general, we can interpret these changes as derived on the human lineage, because the chimpanzee sequence is identical to the inferred human–chimpanzee ancestral sequence. However, additional enhancer experiments using sequence from another mammal or the inferred ancestral sequence will be required to rule out changes acquired on the chimpanzee lineage in a few cases.

Our study revealed that 2xHAR.238 is a forebrain and neural tube enhancer with a more restricted activity pattern in the cortex for the human sequence, compared with chimpanzee (figure 4). It is flanked by two TFs, the zinc finger GLI2 and the repressor TFCP2L1. Another developmental gene, INHBB, is also nearby. The Allen Brain Atlas (http://www.brain-map.org) shows that GLI2 is expressed in the mouse cortex at E11.5 in a pattern consistent with the 2xHAR.238 enhancer (which drives a subset of the GLI2 cortical pattern) and is also expressed in human forebrain early in development through 24 weeks post-conception. Human-specific regulation of GLI2 could potentially have profound developmental effects because this gene is associated with limb defects and forebrain cleavage abnormalities, in addition to being a known mediator of sonic hedgehog signalling in the developing embryo.

2xHAR.114 is likely an enhancer of genes expressed during limb development; the chimpanzee ncHAR enhancer drives expression throughout the limb mesenchyme at E11.5 in transgenic mice, whereas the human sequence drives activity in a limited distal mesenchymal limb domain (figure 5). The 2xHAR.114 enhancer is directly flanked by genes involved in the developing heart (MYLK2, FOXS1). Given the shared pathways between heart and limb development [44], it is possible that these genes are also involved in limb morphogenesis and that the 2xHAR.114 enhancer may influence heart development at a different developmental stage. Other nearby genes are involved in the cytoskeleton, DNA damage response and signal transduction.

The other three ncHARs with suggestive differences between human and chimpanzee exhibit gains of expression with the human sequence. 2xHAR.164 and 2xHAR.170 both drive extensive activity patterns that are consistent across embryos and between the human and chimpanzee sequence. Additionally, only the human 2xHAR.164 and 2xHAR.170 sequences drive expression in the isthmus, a region interposed between the midbrain and hindbrain (figure 6). The isthmus contains the midbrain–hindbrain patterning centre that regulates development of adjacent regions by its production of FGF and WNT ligands. Thus, the gain of human activity in this domain could have important ramifications on development of several structures, such as the cerebellum and catecholaminergic nuclei (e.g. substantia nigra and raphe nuclei). Interestingly, 2xHAR.170 also shows activity in the epithalamus, and development of this structure is also regulated by FGF. Finally, human, but not chimpanzee, HAR25 shows weak eye enhancer activity in the E11.5 mouse (see electronic supplementary material, figure S2). ODZ3 is a developmental gene nearby that could be the target of the HAR25 enhancer.

Further functional studies are needed to validate these observations and to associate changes in enhancer activity to altered expression of specific genes. We anticipate that additional analyses at later developmental stages will likely yield additional insights into expression differences between human and chimpanzee. Our analysis of expression patterns produced by ncHARs considers the chimpanzee sequence as representative of the ancestral state, because the ncHARs exhibit dramatic substitution rate acceleration on the human branch contrasted with much lower substitution rates among chimpanzees and other mammals. However, without directly assaying the activity of additional outgroup mammalian species or inferred ancestral sequences, we cannot exclude the possible, but unlikely, scenario in which the chimpanzee expression profile represents the derived state. With so few human-specific enhancers identified to date, it is difficult to estimate how many ncHARs drive unique expression patterns in humans compared with our ancestors. The recent development of massively parallel reporter assays (MPRAs) [45–47] provides a promising avenue for testing the remaining ncHARs in a variety of cell types at different developmental stages. They will also be useful for identifying specific ncHAR mutations that alter enhancer activity. As larger numbers of ncHARs are tested with MPRAs and lower-throughput functional assays, it may be possible to quantify the total contribution of ncHARs to the evolution of human gene regulation.

What are the next steps for linking genetic changes in ncHARs with detectable molecular phenotypes and organismal traits? One approach is to select individual candidates and undertake low-throughput functional characterizations. These include gel shift assays to validate predictions about changes in TF binding, *in situ* hybridizations in human and chimpanzee tissues to look for overlap between native expression patterns of the predicted target gene and observed enhancer activity of the ncHAR, and genetic studies (e.g. in model organisms) to identify molecular and organismal phenotypes associated with knock-out of the ncHAR, as well as over-expression, knock-down or knock-out of the target gene. These experiments are costly and time-consuming.

Fortunately, computational analyses and high-throughput genomics experiments can help to screen many ncHAR candidates in parallel. For example, human and non-human primate cell lines can be subjected to functional genomics experiments that provide information about human-specific chromatin states (e.g. ChIP-seq for enhancer-associated histone modifications), enhancer–promotor associations (e.g. chromatin conformation capture), and RNA expression patterns (e.g. RNA-seq) in the specific cell types in which ncHARs are hypothesized to function as enhancers. The resulting human-specific functional genomics signatures can then be used to prioritize ncHARs for functional studies. Additionally, comparative genomics and population genomics provide useful information for linking ncHARs to human-specific traits. For instance, comparisons with archaic hominin ncHAR sequences suggest that about 8% of mutations in ncHARs occurred in the past 1 Myr [48], and analyses of diverse human genomes show that approximately 10% of ncHARs contain polymorphisms that are unique to a single world population (L. Arbiza 2013, personal communication). This information about the timing and geographical distribution of ncHAR mutations can then be correlated with the fossil record, environmental conditions and traits that differ between modern human populations to develop testable hypotheses about the role of each ncHAR enhancer in human evolution.

Thus, significant work lies ahead before we will understand the precise mechanisms through which ncHARs contribute to human-specific biology. However, it is now clear that many ncHARs function as developmental enhancers and that human mutations in these conserved non-coding sequences have the potential to alter developmental gene regulation in profound ways. With the aid of computational and genomic approaches, we can now pinpoint ncHARs and specific ncHAR mutations that are the most promising candidates for functional studies of enhancer evolution in the human lineage.

## 4. Material and methods

### (a) Data

All analyses were carried out using the hg19 (GRCh37) build of the reference human genome. All data from previous builds of the genome were mapped to hg19 using the *liftOver* tool from the UCSC Kent tools (http://hgdownload.cse.ucsc.edu/admin/jksrc.zip).

We built a set of ncHARs by combining regions identified in similar tests from several different studies. First, we combined the 202 regions identified in Pollard *et al.* [15] with those identified in an updated analysis based on 29 sequenced mammalian genomes [4] to get the 721 ‘Pollard HARs’. We then combined these with the 1356 ‘ANC’ regions identified by Bird *et al.* [16], the 992 ‘HACNS’ regions from Prabhakar *et al.* [17] and the 63 ‘Bush08’ regions found by Bush & Lahn [18]. All of these regions except for the Pollard HARs were defined to be non-coding. We used gene definitions from version 14 of the GENCODE project [24] to filter out accelerated regions that overlapped coding exons (region_type: exon and gene_type: protein_coding). This produced a set of 2649 ncHARs.

Several sets of annotations of genes and genomic regions were used in this study. Mammalian conserved elements (phastConsElements46wayPlacental) [7] were downloaded from the UCSC Table Browser [49], and all coding elements were filtered out using the GENCODE v. 14 genes. A list of 1988 human TFs was taken from a recent genome-wide analysis [50]. We mined gene descriptions from Uniprot [51] and RefSeq [52] for terms related to embryonic and fetal development to create a list of developmentally relevant genes. We took genes with evidence of differential expression between human and chimpanzee from a study of primate heart, liver, and kidney expression using multi-species microarrays [32] and a more recent comparison of gene expression differences across many tissues and species using RNA-seq [33]. We performed gene ontology annotation and known phenotype enrichment analysis on the ncHARs using version 2.0.2 of GREAT [31] with the default settings and using the non-coding mammalian conserved elements or the whole genome as the background. We also classified the ncHARs based on segmentations of the human genome into seven regulatory states based on ENCODE data from six cell lines [30] and common enhancer marks from a range of cell lines (see electronic supplementary material, table S2) available in the UCSC ENCODE Browser [53]. We downloaded all human and mouse developmental enhancer data deposited in the VISTA Enhancer Browser [35] on 8 March 2013.

### (b) Enhancer prediction and prioritization

To prioritize ncHARs for experimental investigation of their enhancer activity, we used EnhancerFinder [34], a recently developed two-step enhancer prediction pipeline. EnhancerFinder integrates DNA sequence variation and functional genomics data from ENCODE in a multi-kernel support vector machine to distinguish human developmental enhancers from the genomic background, as well as to predict the tissue specificity of predicted enhancers. It was trained on experimentally validated human enhancer sequences from the VISTA Enhancer Browser, and in cross-validation studies, EnhancerFinder was shown to perform extremely well at identifying developmental enhancers. Its ability to distinguish enhancer tissue specificity was generally good, but varied from tissue to tissue.

We applied the first step of EnhancerFinder, which identifies general developmental enhancer activity, to a 1.5 kb region centred on each ncHAR. This produced 773 predicted developmental enhancers. We further classified each of these positives by applying the second round of EnhancerFinder, which consists of classifiers that predict the potential for brain, heart, limb and neural tube expression.

To further prioritize the predicted ncHAR enhancers based on evidence that the human and chimpanzee sequences might drive different expression patterns, we performed an analysis of TFBS divergence between the human and chimpanzee sequences [39]. We compared the number of TFBS motif hits for the 220 vertebrate non-redundant TFs in the April 2012 release of TRANSFAC [54] between the human and chimpanzee versions of ncHARs. We also identified nearby developmentally relevant genes and compared their known functions and expression patterns with the EnhancerFinder tissue specificity predictions. Considering all these data, we manually selected 30 ncHARs for validation with enhancer assays, including one negative control ncHAR that was not predicted to be a developmental enhancer.

### (c) Mouse transgenic enhancer assays

We evaluated the ability of the human and chimpanzee DNA sequences for 29 ncHARs to drive consistent expression patterns during development (see electronic supplementary material, table S4). These assays were performed in transient transgenic mouse embryos generated by pronuclear injections of expression constructs into FVB embryos (Cyagen Biosciences). DNA sequences from either human or chimpanzee were inserted upstream of a minimal promoter and a *LacZ* reporter gene. The DNA sequences were amplified from bacterial artificial chromosomes obtained from the BacPac resource at CHORI. The primers used for each assay are listed in the electronic supplementary material, table S5. Embryos were collected and stained for *LacZ* expression at E11.5.

Following the annotation policies of the VISTA Enhancer Browser [35], we required that consistent spatial expression patterns be present in three or more embryos in order for the region to be considered an enhancer. For the analysis of general enhancer activity, we combined the human and chimpanzee embryos to make activity calls. We divided the validated enhancers into two categories. ‘High confidence’ enhancers are those for which the expression patterns *within* larger expression domains, such as the forebrain, were consistent across many embryos. ncHARs that consistently produced expression in a high level domain, but for which the patterns within the domain varied across embryos were classified as ‘suggestive’. We required at least three embryos with consistent expression within each species and consistent differences between them in high confidence domains for an ncHAR to be classified as a ‘suggestive’ difference between human and chimpanzee. For 2xHAR.114, we compared forelimb activity by calculating the fraction of dark pixels (stained) along a line drawn from the tip of the limb bud to where it met the torso. All embryos with staining are shown in electronic supplementary material, figure S1 and in our searchable online database (http://lighthouse.ucsf.edu/enhancerbrowser/).
